# Functional Interaction Between GABAergic Neurons in the Ventral Tegmental Area and Serotonergic Neurons in the Dorsal Raphe Nucleus

**DOI:** 10.3389/fnins.2022.877054

**Published:** 2022-05-19

**Authors:** Sheikh Mizanur Rahaman, Srikanta Chowdhury, Yasutaka Mukai, Daisuke Ono, Hiroshi Yamaguchi, Akihiro Yamanaka

**Affiliations:** ^1^Department of Neuroscience II, Research Institute of Environmental Medicine, Nagoya University, Nagoya, Japan; ^2^Department of Neural Regulation, Nagoya University Graduate School of Medicine, Nagoya, Japan; ^3^Department of Medicine, Columbia University College of Physicians and Surgeons, New York, NY, United States

**Keywords:** serotonin, GABA, optogenetics, electrophysiology, ventral tegmental area, raphe nucleus

## Abstract

GABAergic neurons in the ventral tegmental area (VTA) have brain-wide projections and are involved in multiple behavioral and physiological functions. Here, we revealed the responsiveness of Gad67+ neurons in VTA (VTA_Gad67+_) to various neurotransmitters involved in the regulation of sleep/wakefulness by slice patch clamp recording. Among the substances tested, a cholinergic agonist activated, but serotonin, dopamine and histamine inhibited these neurons. Dense VTA_Gad67+_ neuronal projections were observed in brain areas regulating sleep/wakefulness, including the central amygdala (CeA), dorsal raphe nucleus (DRN), and locus coeruleus (LC). Using a combination of electrophysiology and optogenetic studies, we showed that VTA_Gad67+_ neurons inhibited all neurons recorded in the DRN, but did not inhibit randomly recorded neurons in the CeA and LC. Further examination revealed that the serotonergic neurons in the DRN (DRN_5–HT_) were monosynaptically innervated and inhibited by VTA_Gad67+_ neurons. All recorded DRN_5–HT_ neurons received inhibitory input from VTA_Gad67+_ neurons, while only one quarter of them received inhibitory input from local GABAergic neurons. Gad67+ neurons in the DRN (DRN_Gad67+_) also received monosynaptic inhibitory input from VTA_Gad67+_ neurons. Taken together, we found that VTA_Gad67+_ neurons were integrated in many inputs, and their output inhibits DRN_5–HT_ neurons, which may regulate physiological functions including sleep/wakefulness.

## Introduction

The VTA is involved in myriad behavioral and physiological functions, such as sleep/wakefulness, motivation, reward prediction error, aversion, reinforcement learning, and addiction (Schultz et al., 1997; Fields et al., 2007; Cohen et al., 2012; Polter and Kauer, 2014; Sun et al., 2017; Bouarab et al., 2019; Zell et al., 2020). It is now well documented that the VTA is composed of heterogeneous neuron types, containing primarily dopamine (DA) neurons (∼65%), but also non-DA neurons, including GABAergic (∼30%) and glutamatergic (∼5%) neurons (Nair-Roberts et al., 2008; Margolis et al., 2012; Pignatelli and Bonci, 2015). γ-aminobutyric acid (GABA), a major inhibitory neurotransmitter in the adult mammalian brain, is synthesized from glutamate by a catalytic enzyme, glutamic acid decarboxylase (GAD). Two isoforms of GAD, GAD67 (also known as GAD1), and GAD65 (also known as GAD2), are found in mammals and are derived from two genes (Erlander et al., 1991). GABAergic neurons in the VTA have profound inhibitory control of the local DA neurons (Tan et al., 2012; Simmons et al., 2017), and they also send projections outside the VTA (Bouarab et al., 2019; Chowdhury et al., 2019). Thus, it is not surprising that inhibitory VTA neurons are involved in multiple behavioral processes, such as innate defensive behavior (Zhou et al., 2019), aversive behavior (Tan et al., 2012; Li et al., 2019), discouragement to get the reward (van Zessen et al., 2012; Pan et al., 2013; Shields et al., 2021), and drug abuse (Nugent et al., 2007; Nugent and Kauer, 2008; Guan and Ye, 2010; Niehaus et al., 2010). Recently, we and others found that GABAergic neurons in the VTA are also critically involved in sleep/wakefulness regulation (Chowdhury et al., 2019; Yu et al., 2019). Although the behavioral and physiological importance of these inhibitory VTA neurons has been gradually revealed, it is still unknown how these neurons are regulated by other neurons, and how that ultimately influences the regulation of various functions. Moreover, our understanding of the major brain areas and cell types regulated by GABAergic neurons from the VTA remain incomplete.

The dorsal raphe nucleus (DRN) in the brainstem also comprises multiple cell types (Monti, 2010a), including serotonergic (5-HT), glutamatergic, GABAergic, and DA neurons (Monti, 2010b; Huang et al., 2019). Among them, 5-HT neurons make up around half of the total neuronal population in the DRN (Descarries et al., 1982). DRN_5–HT_ neurons have multiple physiological functions, including stress and anxiety-like behavior (Hale et al., 2012; Ohmura et al., 2014), mood (Cools et al., 2008), patience and impulsivity (Miyazaki et al., 2012), locomotor activity (Gately et al., 1985; Eagle et al., 2009; Correia et al., 2017), and sleep/wakefulness regulation (Monti, 2010a,b; Moriya et al., 2021). GABAergic neurons are another cell type in the DRN, and they regulate 5-HT neuron activity through a feedforward inhibition (Geddes et al., 2016; Huang et al., 2017; Zhou et al., 2017). The DRN GABAergic population itself is involved in the regulation of energy expenditure (Schneeberger et al., 2019), feeding (Nectow et al., 2017), depressive-like behavior (Xiao et al., 2017), movement control (Seo et al., 2019), real-time place preference (McDevitt et al., 2014), and aversion (Li et al., 2016). Thus, to better understand the cellular underpinnings that lead to such a vast array of behavior and physiology, it is critical to clarify the neural circuitry that regulates these 5-HT and GABAergic neurons in the DRN.

DRN_5–HT_ neurons receive monosynaptic input from the VTA, as was reported in a study using the recombinant rabies virus for retrograde tracing (Weissbourd et al., 2014). And the GABAergic neurons of the VTA have distal projections to different areas of the brain, including to the DRN (Brown et al., 2012; Root et al., 2014; Chowdhury et al., 2019; Li et al., 2019). Here, we searched for evidence of a functional innervation from VTA_Gad67+_ neurons to the CeA, DRN, and LC. Among the neurons in these regions, only those in the DRN were found to be functionally connected to the VTA. Combining optogenetics with electrophysiological recordings, we found that both the DRN_5–HT_ and DRN_Gad67+_ neurons were directly innervated and inhibited by the VTA_Gad67+_ neurons. We also discovered that the DRN_5–HT_ neurons were inhibited by local GABAergic neurons. Furthermore, we explored how VTA_Gad67+_ neurons are modulated by the neurotransmitters serotonin, noradrenaline, dopamine, histamine, carbachol, and orexin A, which are all involved in sleep/wakefulness regulation.

## Materials and Methods

### Animal Usage

All experimental protocols and animal studies were performed in accordance with the approved guidelines of the Institutional Animal Care and Use Committees of the Research Institute of Environmental Medicine, Nagoya University, Japan (Approval numbers: R210096 and R210729). All efforts were made to decrease the usage of animals and to minimize the affliction and pain of animals. Animals were maintained on a 12-h light dark cycle (light period: 08:00–20:00; dark period: 20:00–08:00), with free access to food and water.

### Animals

Adult male and female mice between 8 and 16 weeks of age were used in this study. To specifically label the Gad67-positive neural population in the VTA we used Gad67-Cre mice [Gad1tm2(cre)Tama]. To visualize DRN_5–HT_, we first bred tryptophan hydroxylase 2 (Tph2)–tetracycline transactivator (tTA) mice [Tg(Tph2-tTA)1Ahky] with tetracycline operator sequence (TetO) Yellow-Cameleon Nano50 (YC) mice (Actb^TM 2^.^1Kftnk^) to generate Tph2-tTA;TetO-YC bigenic mice in which Tph2 [which encodes an enzyme that synthesizes 5-HT (Walther et al., 2003)] promoter drives tTA (tetracycline-controlled transactivator) expression in 5-HT neurons. tTA then binds to the TetO sequence and drives expression of YC in 5-HT neurons (Kanemaru et al., 2014; Ohmura et al., 2014; Chowdhury and Yamanaka, 2016; Mukai et al., 2020). These bigenic mice were then crossed with Gad67-Cre mice to finally generate Gad67-Cre; Tph2-tTA; TetO YC trigenic mice. To generate Gad67-Cre; Tph2-tTA bigenic mice, we bred Gad67-Cre mice with Tph2-tTA mice.

### Adeno-Associated Virus Purification and Production

Adeno-associated virus (AAV) vectors were produced using the AAV Helper-Free system (Agilent Technologies, Inc., Santa Clara, CA, United States). The virus purification method was modified from a published protocol (Inutsuka et al., 2016). pAAV plasmid including a gene of interest, pHelper and pAAV-RC (serotype 9) were mixed with 0.3 M CaCl_2_, followed by the addition of 2× HEPES buffered saline in a dropwise manner with this solution during continuous vortex mixing. For transfection, the mixture of calcium-phosphate with DNA was added to cultured HEK-293 cells in Dulbecco’s Modified Eagle’s Medium-high glucose medium (DMEM) (Sigma-Aldrich, Merck, Darmstadt, Germany). After 3 days, transfected cells were collected and centrifuged. The pellet was suspended with phosphate buffered saline (PBS). The cell suspension was then freeze-thawed four times with the following cycle: frozen at −80°C for 15 min, incubated at 37°C for 10 min and vortexed at room temperature (RT) for 1 min. Then, benzonase nuclease (0.025 U, Merck, Darmstadt, Germany) was added to the cell suspension solution and it was incubated at 37°C for 30 min. The solution was centrifuged twice at 16,000 rpm for 10 min. The supernatant was used as the AAV-containing solution. Quantitative PCR was performed to measure the number of particles of purified AAV. The AAV was aliquoted and stored at −80°C until use.

### Adeno-Associated Virus Injection Using a Stereotaxic Frame

At 8–16 weeks of age, male and female mice (Gad67-Cre, Gad67-Cre;Tph2-tTA;TetO YC and Gad67-Cre;Tph2-tTA) received either unilateral (in DRN) or bilateral (in VTA) injection of AAV9-CMV-FLEX-hrGFP (200 nl, 7.45 × 10^12^ copies/ml), AAV9-CMV-FLEX-ChR2(ET/TC)-EYFP (200 nl, 1.5 × 10^13^ copies/ml), AAV9-CMV-FLEX-tdTomato (200 nl, 2 × 10^12^ copies/ml) or AAV9-TetO(3G) tdTomato (200 nl, 6.6 × 10^11^ copies/ml) under 1.0–1.2% isoflurane (Fujifilm Wako Pure Chemical Corporation, Osaka, Japan) anesthesia (MK-A100W, Muromachi Kikai Co., Fukoaka, Japan). Injection sites were as follows: from bregma -3.3 mm, lateral ±0.5 mm, ventral -4.2 mm for the VTA and from bregma -4.5 mm, lateral 0 mm, ventral -3.1 mm for the DRN.

### Immunohistochemical Study

Mice were deeply anesthetized with isoflurane (Fujifilm Wako Pure Chemical Corporation, Osaka, Japan) and transcardially perfused with 20 ml of ice-cold saline, followed by 20 ml of ice-cold 10% formalin (Fujifilm Wako Pure Chemical Industries, Osaka, Japan). Brains were removed and post-fixed in a 10% formalin solution at 4°C overnight. Brains were then cryoprotected in 30% sucrose in PBS at 4°C for at least 2 days. After that, brains were frozen in embedding OCT compound (Sakura Finetek, Osaka, Japan) at −80°C, and coronal brain slices of 40 or 80 μm thickness were made using a cryostat (CM3050-S, Leica Microsystems K.K., Wetzlar, Germany) at −20°C. Slices were placed in PBS containing 0.02% NaN_3_ at 4°C until staining. For staining, coronal brain slices were washed 3 times with blocking buffer (1% BSA and 0.25% Triton-X in PBS) for 10 min and then incubated with primary antibodies at 4°C overnight. For Gad67 staining, slices were incubated with the primary antibody at 4°C for 2 days. After washing 3 times with blocking buffer for 10 min, slices were then incubated with secondary antibodies for 1 h at RT. For Gad67 staining, slices were incubated with secondary antibody for 2 h at RT. After another 3 washes with blocking buffer, slices were stained with 4 μM DAPI (4′,6-diamidino-2-phenylindole, Wako Pure Chemical Industries, Osaka, Japan) (except for Figure 7B) for 10 min at room temperature. Finally, slices were washed 3 times with blocking buffer and then mounted and examined with a fluorescence microscope (BZ-X710, Keyence, Osaka, Japan) and/or confocal microscope (LSM 710, Zeiss, Jena, Germany). Every fourth coronal brain section of both hemispheres was used for cell counts.

**FIGURE 1:**
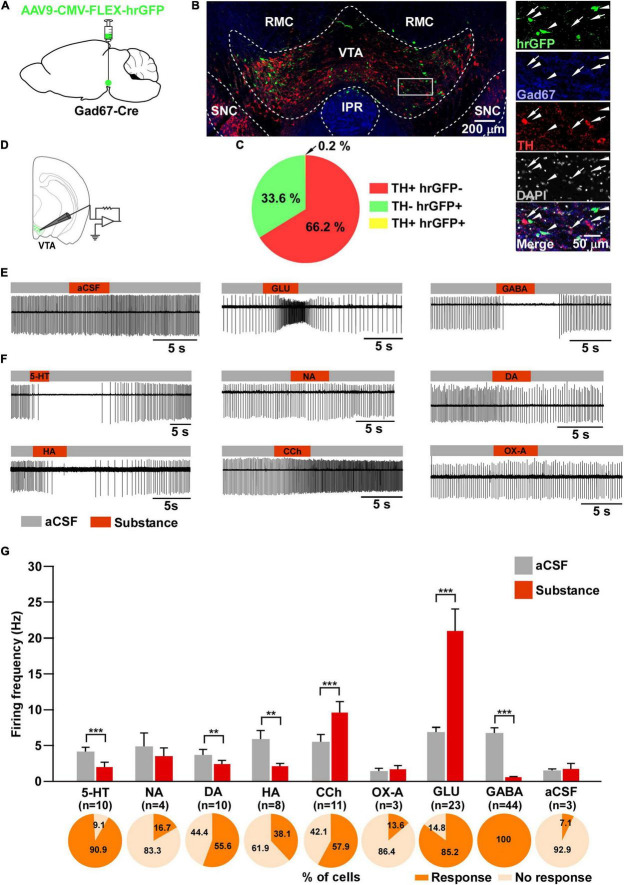
Responsiveness of VTA_Gad67+_ neurons to neurotransmitters. **(A)** Schematic of the AAV injection used to visualize VTA_Gad67+_ neurons. **(B)** Immunostaining shows expression of hrGFP in the VTA. The white rectangular area is magnified on the right side, demonstrating that hrGFP expression is found only in Gad67-positive neurons (white arrowheads) and not in the DA neurons (white arrows). Green (hrGFP), blue (Gad67), red (TH), gray (DAPI), white (merge). RMC, red nucleus magnocellular; IPR, interpeduncular nucleus; rostral; SNC, substantia nigra compacta. **(C)** Pie chart showing the percentage of hrGFP-expressing cells among DA and non-DA neurons in the VTA (*n* = 4 mice). **(D)** Schematic of the patch clamp recording from VTA_Gad67+_ neurons. **(E,F)** Representative traces showing the effect of local application. Gray bars indicate aCSF application and red bars indicate substance application. **(G)** Bar graphs summarize the firing frequency of responsive cells following the application of each substance, and pie charts show the percentage of responsive and non-responsive cells, where *n* indicates the number of responsive cells. Data are shown as means ± SEMs. Statistical analyses were made using two-tailed paired Student’s *t*-test. ** *p* < 0.01, *** *p* < 0.001.

**FIGURE 2:**
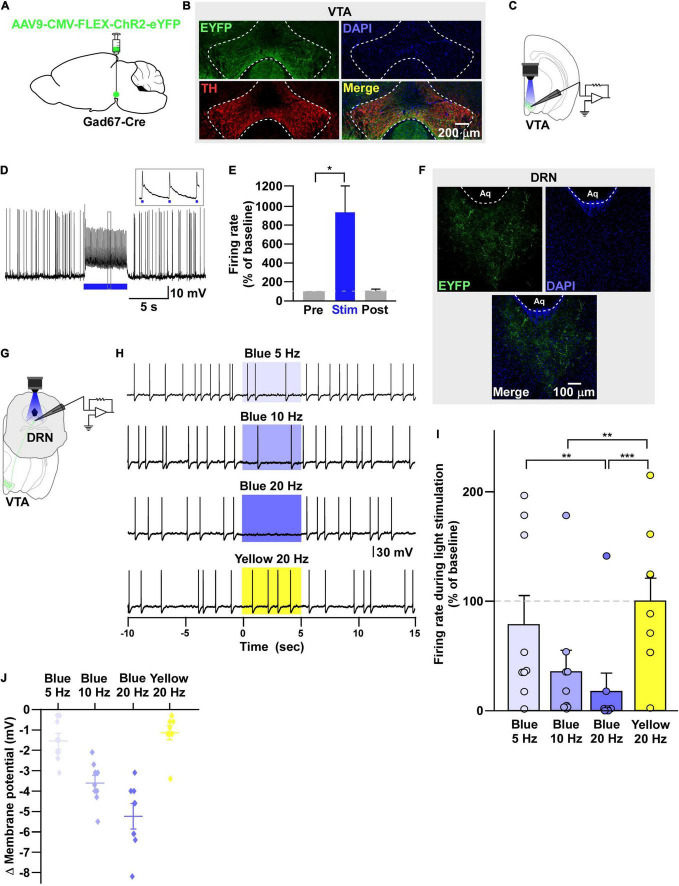
VTA_Gad67+_ neurons inhibit DRN neurons. **(A)** Schematic of the AAV injection used to express ChR2-eYFP in VTA_Gad67+_ neurons. **(B)** Immunostaining shows the expression of ChR2-eYFP in the VTA, which does not overlap with TH expression. Green (EYFP), red (TH), blue (DAPI), yellow (merge). **(C)** Schematic and **(D)** a representative trace of *in vitro* whole-cell current clamp recordings from ChR2-expressing VTA_Gad67+_ neurons. The blue bar indicates the 10-Hz blue light (3.19 mW) pulse, which was applied for 5 s. Inset shows the pulse-generated action potentials. **(E)** Summary of **(D)** (*n* = 8 cells from 5 mice). **(F)** VTA_Gad67+_ nerve terminals in the DRN. Green (EYFP), blue (DAPI), white (merge). Aq, aquaduct. **(G)** Schematic of the setup for recording from DRN neurons. **(H)** Representative traces from the current clamp recordings of neurons in the DRN following activation of VTA_Gad67+_ nerve terminals by applying a 5-, 10-, or 20-Hz stimulation of blue light (3.19 mW) for 5 s. Yellow light (0.8 mW) stimulation (20 Hz) was used as the control. **(I,J)** Summary data of **(H)**, showing that firing rate and membrane potential decreased in a blue light frequency–dependent manner compared with yellow light (*n* = 9 cells from 2 mice). Data are shown as means ± SEMs. Statistical analysis for **(E)** was made using two-tailed paired Student’s *t*-test and for **(I)** were made using one-way repeated measures ANOVA with Tukey’s multiple comparison test. * *p* < 0.05, ** *p* < 0.01, *** *p* < 0.001.

**FIGURE 3:**
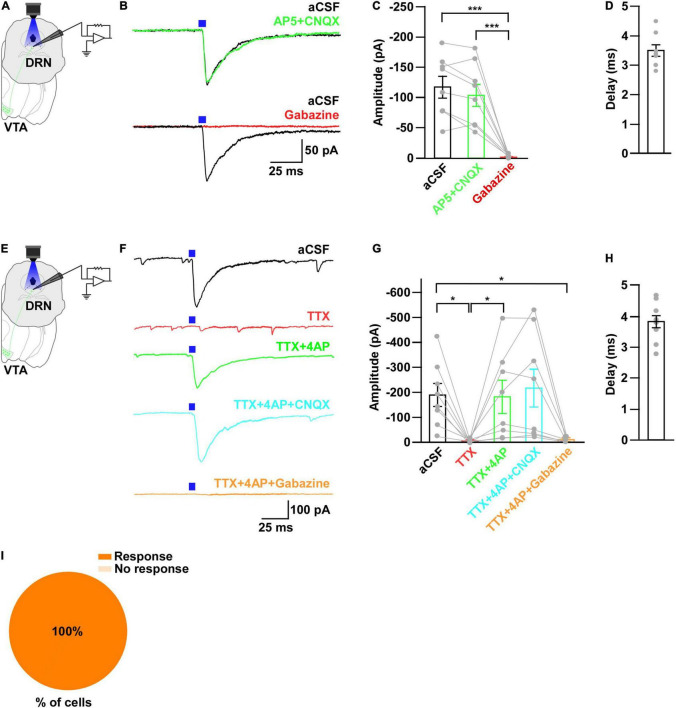
Neurons in the DRN receive monosynaptic GABAergic inputs from VTA_Gad67+_ neurons. **(A,E)** Schematic of the whole-cell voltage clamp setup for recording from neurons in the DRN. **(B)** Blue light pulses (5 ms) induced PSCs in the DRN by using glutamatergic and GABAergic blockers. **(C)** Summary of **(B)** showing the amplitudes of inward currents (*n* = 8 cells 2 mice). **(D,H)** Response delay from light onset (*n* = 8 cells from 2 mice). **(F)** Blue light pulses (5 ms) induced PSCs in neurons in the DRN. **(G)** Summary of **(F)**, showing the amplitudes of inward currents (*n* = 8 cells from 2 mice). **(I)** Pie chart showing the percentage of responsive cells. Data are means ± SEMs. All statistical analyses were made using one-way repeated measures ANOVA with Tukey’s multiple comparison test. * *p* < 0.05, *** *p* < 0.001.

**FIGURE 4:**
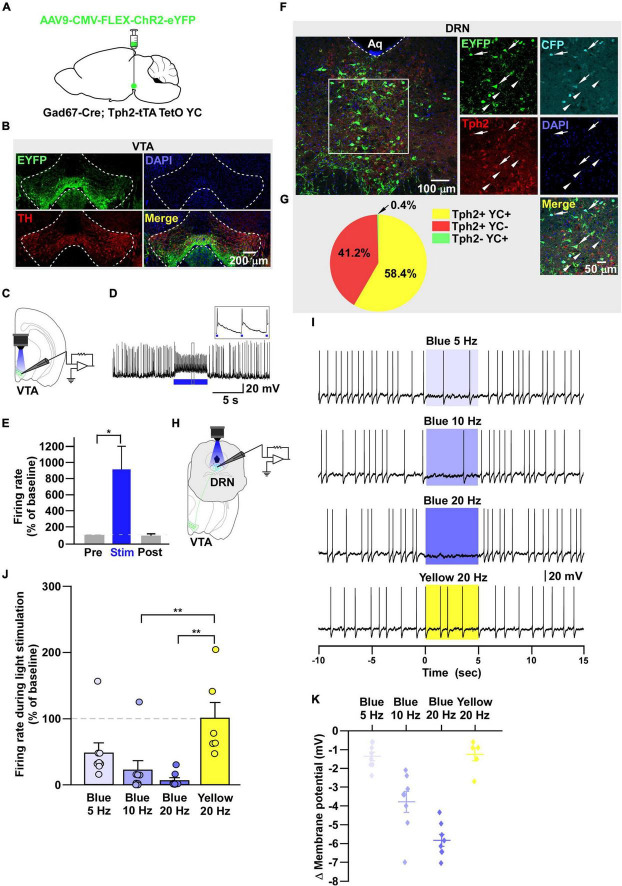
DRN_5–HT_ neurons are inhibited by VTA_Gad67+_ neurons. **(A)** Schematic of the AAV injection used to express ChR2-eYFP in VTA_Gad67+_ neurons. **(B)** Immunostaining shows the expression of ChR2-eYFP in the VTA, indicating there is no overlap with TH-positive cells. Green (EYFP), red (TH), blue (DAPI), yellow (merge). **(C)** Schematic of the setup and **(D)** a representative trace from the *in vitro* whole-cell current clamp recordings from ChR2-expressing VTA_Gad67+_ neurons. Blue bar indicates 10 Hz of blue light (3.19 mW) stimulation for 5 s. Inset shows the pulse generated-action potentials. **(E)** Summary of **(D)** (*n* = 8 cells from 4 mice). **(F)** A DRN-containing coronal brain slice showing the expression of eYFP-positive nerve terminals from VTA_Gad67+_ neurons (arrowheads) and colabeling of CFP- and Tph2-positive 5-HT neurons (arrows). Green (EYFP), cyan (CFP/YC), red (Tph2), blue (DAPI), yellow (merge). Aq, aquaduct. **(G)** Pie chart shows the percentage of YC-expressing cells among 5-HT–positive neurons (*n* = 5 mice). **(H)** Schematic of the setup for recording from DRN_5–HT_ neurons. **(I)** Representative traces of current clamp recordings from DRN_5–HT_ neurons following activation of VTA_Gad67+_ neuronal terminals by applying 5-, 10-, and 20-Hz stimulation of blue light (3.19 mW) for 5 s. Yellow light (0.8 mW) stimulation (20 Hz) was used as the control. **(J,K)** Summary of **(I)**. Firing rates and membrane potentials were decreased in a frequency-dependent manner compared with yellow light (*n* = 8 cells for 5 and 10-Hz blue light; *n* = 9 cells for 20-Hz blue light; *n* = 6 cells for yellow light from 3 mice). Data are shown as means ± SEMs. Statistical analysis for **(E)** was made using two-tailed paired Student’s *t*-test and for **(J)** were made using a one-way ANOVA with Tukey’s multiple comparison test. * *p* < 0.05, *** *p* < 0.001.

**FIGURE 5:**
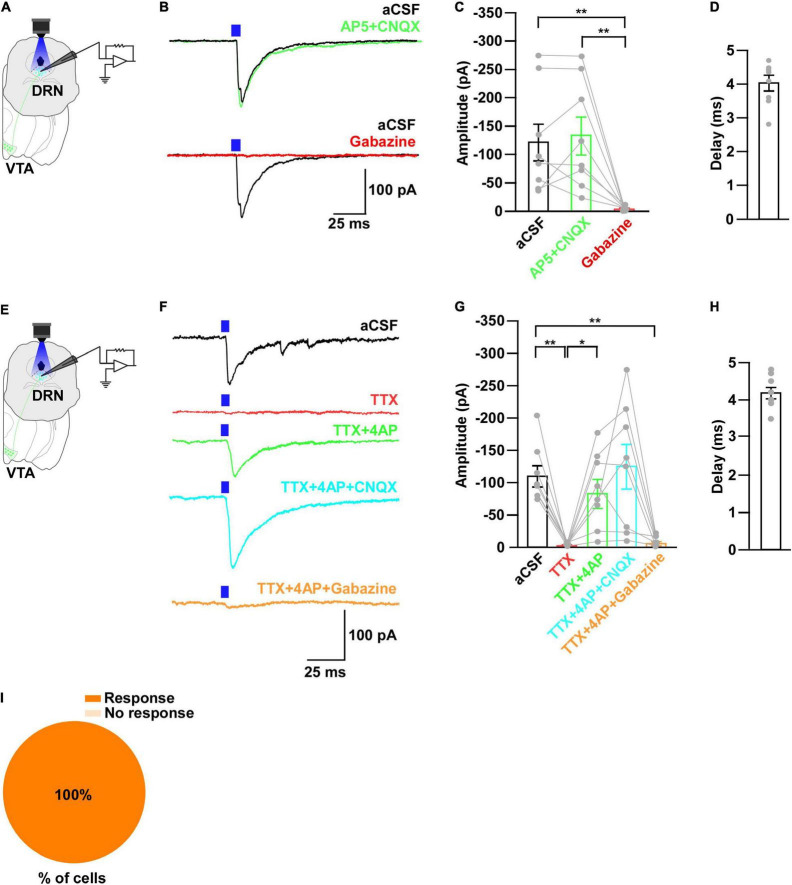
DRN_5–HT_ neurons receive monosynaptic inputs from VTA_Gad67+_ neurons. **(A,E)** Schematic of the setup for whole-cell voltage clamp recording from DRN_5–HT_ neurons. **(B)** Blue light pulses (5 ms) induced PSCs in DRN_5–HT_ neurons by using glutamatergic and GABAergic blockers. **(C)** Summary of **(B)** showing the amplitudes of inward currents (*n* = 8 cells from 3 mice). **(D,H)** Response delay from light onset. **(F)** Blue light pulses (5 ms) induced PSCs in DRN_5–HT_ neurons by using different blockers. **(G)** Summary of **(F)**, showing the amplitudes of inward currents (*n* = 8 cells from 2 mice). **(I)** Pie chart showing the percentage of responsive cells. Data are shown as means ± SEMs. All statistical analyses were made using a one-way repeated measures ANOVA with Tukey’s multiple comparison test. * *p* < 0.05, ** *p* < 0.01.

**FIGURE 6:**
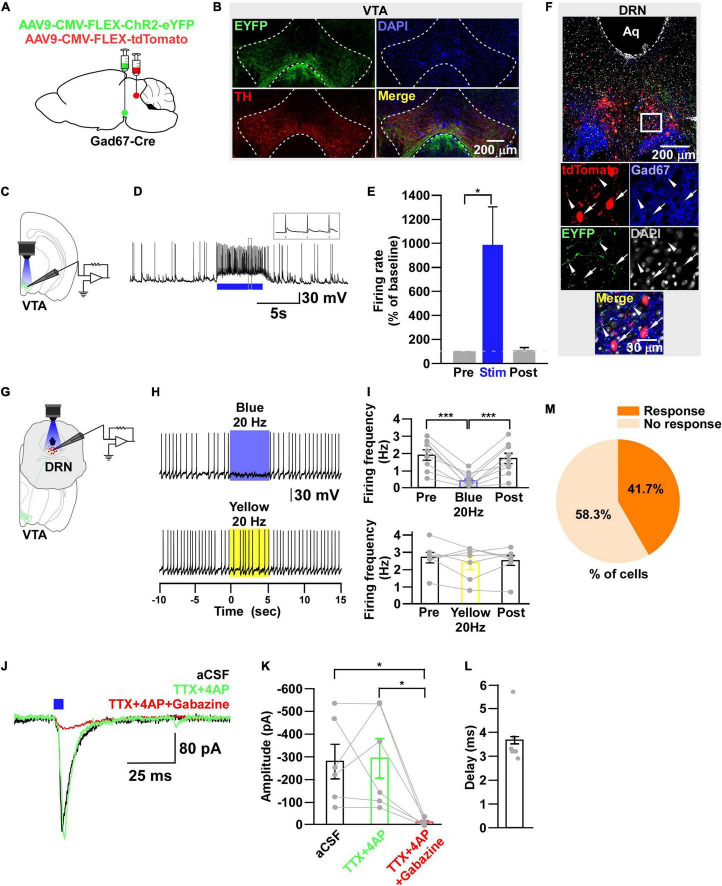
DRN_Gad67+_ neurons are inhibited by VTA_Gad67+_ neurons. **(A)** Schematic of the AAV injection used to express ChR2-eYFP in VTA_Gad67+_ neurons and tdTomato in DRN_Gad67+_ neurons. **(B)** Immunostaining shows expression of ChR2-eYFP in the VTA, which does not overlap with TH-positive cells. Green (EYFP), red (TH), blue (DAPI), yellow (merge). Aq, aquaduct. **(C)** Schematic of the setup and **(D)** a representative trace of *in vitro* whole-cell current clamp recordings from ChR2-expressing VTA_Gad67+_ neurons. Blue bar indicates a 10-Hz blue light (3.19 mW) pulse for 5 s. Inset shows the pulse generated-action potentials. **(E)** Summary of **(D)** (*n* = 7 cells from 3 mice). **(F)** A DRN-containing coronal brain slice showing the expression of eYFP nerve terminals from VTA_Gad67+_ neurons (white arrowheads) and colabeling of tdTomato and Gad67-positive GABA neurons (white arrow). The white square area is magnified in the lower panel. Green (EYFP), blue (Gad67), red (tdTomato), gray (DAPI), yellow (merge). **(G)** Schematic of recording from DR_Gad67+_ neurons. **(H)** Representative traces of current clamp recordings from DR_Gad67+_ neurons and activation of VTA_Gad67+_ nerve terminals by applying a 20-Hz stimulation of blue light (3.19 mW) for 5 s. Yellow light (0.8 mW; 20 Hz) was used as a control. **(I)** Summary of **(H)**, showing firing frequencies comparing pre-, post-, and yellow light stimulation (*n* = 9 cells for blue, *n* = 7 cells for yellow from 3 mice). **(J)** Blue light pulses (5 ms) induced PSCs in DRN_Gad67+_ neurons. **(K)** Summary of **(J)**, showing the amplitudes of inward currents (*n* = 6 cells from 3 mice). **(L)** Response delay from light onset. **(M)** Pie chart showing the percentage of responsive cells. Data are shown as means ± SEMs. Statistical analysis for **(E)** was made using two-tailed paired Student’s *t*-test and for **(I,K)** were made using a one-way repeated measures ANOVA with Tukey’s multiple comparison test. * *p* < 0.05, *** *p* < 0.001.

**FIGURE 7:**
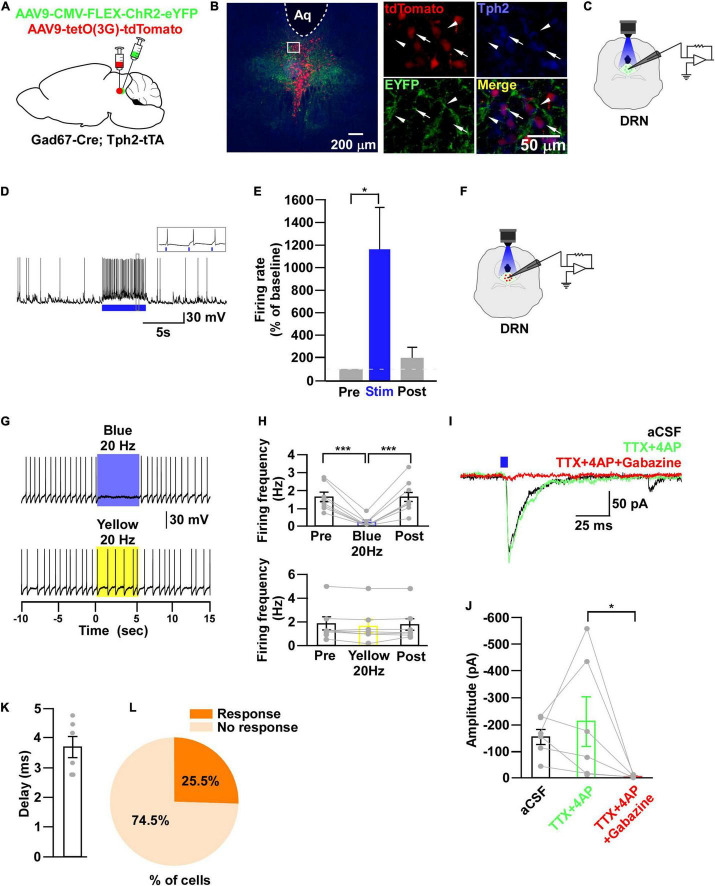
DRN_5–HT_ neurons are inhibited by DRN_Gad67+_ neurons. **(A)** Schematic of the AAV injection used to express ChR2-eYFP in DRN_5–HT_ neurons and tdTomato in DRN_Gad67+_ neurons. **(B)** DRN-containing coronal brain slices show the expression of eYFP (white arrowheads) and the colabeling of tdTomato and Tph2-positive 5-HT neurons (white arrows). The white square area is magnified on the right side. Green (EYFP), red (tdTomato), blue (Tph2), yellow (merge). Aq, aquaduct. **(C)** The schematic setup and **(D)** a representative trace of *in vitro* whole-cell current clamp recordings from ChR2-expressing DRN_Gad67+_ neurons. **(D)** The blue bar below the trace indicates the 10-Hz blue light (3.19 mW) pulse for 5 s. Inset shows the pulse-dependent action potential generation. **(E)** Summary of **(D)** (*n* = 6 cells from 4 mice). **(F)** Schematic of the setup for recording from tdTomato-expressing DRN_5–HT_ neurons. **(G)** Representative traces of current clamp recordings from DRN_5–HT_ neurons and activation of DRN_Gad67+_ nerve terminals by applying a 20-Hz stimulation of blue light (3.19 mW) for 5 s. Yellow light (0.8 mW; 20 Hz) was used as a control. **(H)** Summary of **(G)**, showing the firing frequencies compared with pre-, post-, and yellow light stimulation (*n* = 8 cells for blue, *n* = 7 cells for yellow from 3 mice). **(I)** Blue light pulses (5 ms) induced PSCs in DRN_5–HT_ neurons by using different blockers. **(J)** Summary of **(I)** showing the amplitudes of inward currents (*n* = 6 cells from 2 mice). **(K)** Response delay from light onset. **(L)** Pie chart showing the percentage of responsive cells. Data are shown as means ± SEMs. Statistical analysis for **(E)** was made using two-tailed paired Student’s *t*-test and for **(H,J)** were made using a one-way repeated measures ANOVA with Tukey’s multiple comparison test. * *p* < 0.05, *** *p* < 0.001.

### Antibodies

Primary and secondary antibodies were diluted in the blocking buffer at the following concentrations: anti–tyrosine hydroxylase (TH) rabbit antibody (AB152, Millipore, Darmstadt, Germany) 1/1000, anti-Gad67 mouse antibody (MAB5406, Millipore, Darmstadt, Germany) 1/250, anti-Tph2 sheep antibody (AB1541, Millipore, Darmstadt, Germany) 1/250, anti-green fluorescent protein (GFP) chicken antibody (Aves labs, Inc., Davis, CA, United States) 1/1000, CF 350-conjugated anti-sheep antibody (20148, Biotium, Fremont, CA, United States) 1/1000, CF 488-conjugated anti-chicken antibody (20166, Biotium, Fremont, CA, United States) 1/1000, CF 594-conjugated anti-rabbit antibody (20152, Biotium, Fremont, CA, United States) 1/1000, CF 594-conjugated anti-sheep antibody (20156, Biotium, Fremont, CA, United States) 1/1000 and CF 647-conjugated anti-mouse antibody (20046, Biotium, Fremont, CA, United States) 1/1000.

### Electrophysiological Recording

Adult male and female mice between 10 and 24 weeks of age were used for electrophysiological recording experiments. After 3–4 weeks of AAV injection, mice were deeply anesthetized using isoflurane (Fujifilm Wako Pure Chemical Corporation, Osaka, Japan) and decapitated. Brains were rapidly isolated and chilled in ice-cold bubbled (95% O_2_ and 5% CO_2_) cutting solution (in mM: 110 K-gluconate, 15 KCl, 0.05 EGTA, 5 HEPES, 26.2 NaHCO_3_, 25 glucose, 3.3 MgCl_2_, 0.0015 3-[(±)-2-carboxypiperazin-4-yl)-propyl-1-phosphonic acid (CPP)]. Coronal brain sections of 300-μm thickness were made using a vibratome (VT-1200S, Leica, Tokyo, Japan). The slices were incubated in a bubbled (95% O_2_ and 5% CO_2_) bath solution (in mM: 124 NaCl, 3 KCl, 2 MgCl_2_, 2 CaCl_2_, 1.23 NaH_2_PO_4_.2H_2_O, 26 NaHCO_3_, 25 glucose) at 35°C for 30 min following another 30-min incubation at RT in the same solution. After the incubation period, the brain slice was placed in a recording chamber (RC-26G, Warner Instruments, Hamden, CT, United States) that was perfused with a bubbled (95% O_2_ and 5% CO_2_) bath solution at a rate of 1.5 ml/min using a peristaltic pump (Miniplus3, Gilson, United States). An infrared camera (C3077-78, Hamamatsu Photonics, Hamamatsu, Japan) was installed in an upright fluorescence microscope (BX51WI, Olympus, Tokyo, Japan), together with an electron multiplying charge-coupled device camera (Evolve 512 delta, Photometrics, Tucsaon, AZ, United States), and both images were visualized on separate monitors. Glass micropipettes were prepared from borosilicate glass capillaries (GC150-10, Harvard Apparatus, Cambridge, MA, United States) using a horizontal puller (P1000 Sutter Instrument, Novato, CA, United States), maintaining a pipette resistance of 4–8 MΩ. During recording from ChR2-eYFP–expressing VTA_Gad67+_ and DRN_Gad67+_ neurons, patch pipettes were filled with a KCl-based internal solution (in mM: 145 KCl, 1 MgCl_2_, 10 HEPES, 1.1 EGTA, 2 Mg-ATP, 0.5 Na-GTP; brought to pH 7.3 with KOH), with an osmolality between 280 and 290 mOsm. A K-gluconate–based internal solution (in mM: 138 K-gluconate, 10 HEPES, 8 NaCl, 0.2 EGTA, 2 Mg-ATP, 0.5 Na_2_-GTP; brought to pH 7.3 with KOH), with an osmolality between 285 and 290 mOsm was used while recording from CeA, DRN and LC non-specific neurons, as well as from DRN_5–HT_, DRN_non–5–HT_, and DR_Gad67+_ neurons in current clamp mode and from a few DRN_non–5–HT_ neurons in loose cell-attached mode. After confirming that the cell expressed green (hrGFP/eYFP), red (tdTomato) or YC, the pipette was moved toward the cell. A positive pressure was applied manually in the patch pipette. This pressure was released when the tip of the pipette touched the cell membrane and a gigaseal was formed. Then, the patch membrane was ruptured by briefly applying strong suction to form a whole-cell configuration. During recording, electrophysiological properties were continuously observed using the Axopatch 200B amplifier (Axon Instrument, Molecular Devices, San Jose, CA, United States). Output signals were low-pass filtered at 5 kHz and digitized at a 10-kHz sampling rate. Patch clamp data were recorded through an analog-to-digital converter (Digidata 1550A, Molecular Devices, San Jose, CA, United States) using pClamp 10.7 software (Molecular Devices, San Jose, CA, United States). Blue and yellow light (with a wavelength of 475 ± 17.5 and 575 ± 12.5 nm, respectively) was generated by a light source that used a light-emitting diode (Spectra light engine, Lumencor, Beaverton, OR, United States) and was directed to the microscope stage with a 1.0 cm diameter optical fiber. Coronal brain slices were illuminated through the objective lens of the fluorescence microscope. In whole-cell voltage clamp recordings at a −60 mV holding potential, QX-314 (1 mM) was added to the KCl-based pipette solution. A six-channel perfusion valve controller (VC-6, Warner Instruments, Holliston, MA, United States) was used for local substance perfusion.

### Quantification and Statistical Analysis

Immunostaining data were quantified, analyzed with ImageJ (United States National Institutes of Health) and BZ-X Analyzer (Keyence BZ-X710 microscope, Itasca, IL, United States) software. Electrophysiological data were analyzed by Clampfit10 (Molecular Devices, San Jose, CA, United States). Statistical analyses were performed using OriginPro 2020 software. Graphs were generated using Canvas X (Canvas GFX Inc., Boston, MA, United States). Simple comparisons of the means and standard errors of the mean (SEMs) were performed using a Student’s *t*-test. Multiple comparisons of means and SEMs were performed by a one-way ANOVA (with/without repeated measures) analysis followed by Tukey’s test. *p* values < 0.05 were considered significant.

## Results

### Responsiveness of VTA_Gad67+_ Neurons to Various Neurotransmitters

To visualize VTA_Gad67+_ neurons, Gad67-Cre mice were bilaterally injected with a Cre-dependent AAV expressing humanized renilla GFP (hrGFP) into the VTA (Figure 1A). Two weeks after injection, many hrGFP-expressing neurons were observed in the VTA (Figure 1B). We used every fourth section of 40 μm coronal brain slices where double immunostaining for Gad67 (a marker of GABAergic neurons) and TH (a marker of DA neurons) confirmed that most of the hrGFP-expressing neurons in the VTA colocalized with Gad67, but not with TH (Figure 1B). Of all the VTA neurons, 66.2 ± 2.3% were TH-positive, 33.6 ± 2.3% were hrGFP-positive and only 0.2 ± 0.1% were co-labeled with TH and hrGFP (Figure 1C). We counted 1140 ± 58 neurons per animal (*n* = 4 mice).

Next, we performed slice patch clamp recording to assess the activity of VTA_Gad67+_ neurons upon application of various substances. Coronal brain sections of hrGFP-expressing VTA_Gad67+_ neurons from Gad67-Cre mice were prepared (Figure 1D). By using a six-channel perfusion valve controller, substances were sequentially and locally applied for 4 s under a loose cell-attached mode recording. To minimize mechanical or any other indirect effects on the recording cell by local application of the substance, artificial cerebrospinal fluid (aCSF) was applied before and after substance application (Figures 1E,G). At first, glutamate (Glu, 100 μM) and GABA (100 μM) (Figures 1E,G) were applied to confirm responsiveness and positioning of the local application. After that, serotonin (5-HT, 50 μM), noradrenaline (NA, 100 μM), dopamine (DA, 100 μM), histamine (HA, 100 μM), carbachol (CCh, 100 μM), and orexin A (OX-A, 100 nM) were applied in random order. We found that 5-HT, DA and HA application significantly reduced, whereas CCh increased, the firing rate (Figures 1F,G). Surprisingly, the wake-promoting neurotransmitters, NA and OX-A had no significant effect on firing frequency (Figures 1F,G). We then counted the fraction of cells that responded to substance application. We considered cells to be responsive if they showed a ≥20% increase or decrease in firing frequency during the 4 s substance application compared with before application. We found responses in 10/11 (90.9%) cells with 5-HT, 4/24 (16.7%) cells with NA, 10/18 (55.6%) cells with DA, 8/21 (38.1%) cells with HA, 11/19 (57.9%) cells with CCh, 3/22 (13.6%) cells with OX-A, 23/27 (85.2%) cells with Glu, 44/44 (100%) cells with GABA and 3/42 (7.1%) cells with aCSF (Figure 1G, pie charts). These results indicated that VTA_Gad67+_ neurons responsiveness to substances was not uniform.

### Non-specific Recording From the Downstream Target of VTA_Gad67+_ Neurons

Next, we sought to reveal the downstream target neurons of VTA_Gad67+_ neurons. We bilaterally injected a Cre-dependent AAV carrying the blue light–gated cation channel channelrhodopsin-2 (E123T/T159C) (ChR2) (Berndt et al., 2011) into the VTA of Gad67-Cre mice (Figure 2A). Immunohistochemical study confirmed that ChR2 fused with eYFP was expressed exclusively in the VTA (Figure 2B). *In vitro* slice patch clamp recording from ChR2-expressing VTA_Gad67+_ neurons confirmed that a 10-Hz blue light pulse depolarized and significantly increased the spontaneous firing frequency by 919 ± 277% compared with pre-stimulation values (Figures 2C–E). VTA_Gad67+_ neurons densely projected to the distal part of the brain, including to the CeA, DRN, and LC, consistent with our previous study (Figure 2F; Supplementary Figures 1A,B; Chowdhury et al., 2019). We then recorded from neurons in the CeA, DRN, and LC by stimulating the VTA_Gad67+_ nerve terminals. Current clamp recording showed that only the recorded neurons of the DRN were hyperpolarized and there was a complete cessation of spontaneous firing following a 20-Hz blue light stimulation, whereas activity in CeA or LC neurons was not affected (Supplementary Figures 1C–K). We then focused on the DRN—we randomly selected cells in the DRN and applied different light frequencies, i.e., 5-, 10-, and 20-Hz blue light stimulation in whole-cell current clamp mode (Figure 2G). We found that blue light decreased the firing rate and hyperpolarized the membrane potential in a frequency-dependent manner. However, stimulation with the 20-Hz yellow light showed no effect on firing frequency (Figures 2H–J).

To further reveal the mechanism of action, we recorded from neurons in the DRN in whole-cell voltage clamp mode at a -60 mV holding potential. Activation of VTA_Gad67+_ nerve terminals in the DRN induced post-synaptic currents (PSCs) in response to a 5-ms blue light pulse (Figures 3A,B). These light-induced PSCs were blocked by a GABA_A_ receptor antagonist gabazine (10 μM), but not by AP5 (50 μM) and CNQX (20 μM), which are antagonists for the NMDA (*N*-methyl-D-aspartic acid) and AMPA (α-amino-3-hydroxy-5-methyl-4-isoxazolepropionic acid) glutamate receptor types, respectively (Figures 3B,C). The mean response delay from light onset was 3.5 ± 0.2 ms (Figure 3D). To rule out the effect of indirect input, we applied tetrodotoxin (TTX, 1 μM), a voltage-gated sodium channel blocker, which blocked the light-induced PSCs (Figures 3E–G). The combination of TTX and 4AP (4-aminopyridine, 1 mM), a voltage-gated potassium channel blocker, rescued the light-induced PSCs. These were again blocked by gabazine but not by CNQX (Figures 3F,G). We found the mean response delay was 3.8 ± 0.2 ms (Figure 3H). We recorded 35 cells from 5 mice (included in Figure 2I and Supplementary Figure 1H), where all the cells (35/35, 100%) responded to blue light stimulation (Figure 3I). Together, these results confirmed that DRN neurons were directly innervated and inhibited by VTA_Gad67+_ neurons through the neurotransmitter GABA.

### DRN_5–HT_ Neurons Are Directly Inhibited by VTA_Gad67+_ Neurons

It has been reported that there are different subtypes of neurons in the DRN (Huang et al., 2019). Among them, 5-HT neurons are a major population and known to be involved in many physiological functions. Thus, the DRN neurons we recorded in the earlier experiment might have been serotonergic. To confirm this, we generated trigenic mice—Gad67-Cre;Tph2-tTA;TetO YC—in which tTA was exclusively expressed in 5-HT neurons under the control of the Tph2 promoter (Gossen et al., 1995). tTA binds to the TetO sequence and induces the YC expression (Tanaka et al., 2012). We then bilaterally injected a Cre-dependent AAV carrying ChR2 into the VTA of trigenic mice (Figure 4A). Immunostaining results confirmed that ChR2 fused with eYFP was exclusively expressed in the VTA (Figure 4B). *In vitro* slice patch clamp recording from ChR2-expressing VTA_Gad67+_ neurons confirmed that these neurons were depolarized by a 10-Hz blue light pulse, and this significantly increased the spontaneous firing frequency by 908 ± 289% compared with pre-stimulation values (Figures 4C–E). Since YC is composed of yellow fluorescent protein (YFP) and cyan fluorescent protein (CFP), we could clearly isolate the expression of YFP versus CFP by confocal imaging. Although CFP co-labeled with YFP, the nerve terminals from VTA_Gad67+_ neurons expressed only YFP and not CFP (Figure 4F, white arrowheads). We stained every fourth 40 μm section of DRN-containing brain slices with an anti-Tph2 antibody to label 5-HT neurons. Colocalization of YC and TPH2-positive cell bodies confirmed that 5-HT neurons expressed YC (Figure 4F, white arrows). Cell counting indicated that 58.4 ± 2.6% of cells were both TPH2- and YC-positive, 41.2 ± 2.7% were only positive for TPH2 and 0.4 ± 0.1% were only YC-positive (Figure 4G). We then recorded from the YC-expressing DRN5-HT neurons by stimulating VTA_Gad67+_ nerve terminals (Figure 4H). In whole-cell current clamp recording, we found that a 5-, 10- and 20-Hz blue light pulse decreased the firing rate and hyperpolarized the membrane potential in a frequency-dependent manner. However, stimulation with a 20-Hz yellow light had no effect on firing frequency (Figures 4I–K).

Next, to further clarify the mechanism of action, we recorded from the 5-HT neurons in whole-cell voltage clamp mode at a -60 mV holding potential. Activation of nerve terminals from VTA_Gad67+_ neurons in the DRN induced PSCs in response to a 5-ms blue light pulse (Figures 5A,B). These light-induced PSCs were blocked by gabazine (10 μM), but not by combined AP5 (50 μM) and CNQX (20 μM) (Figures 5B,C). The mean response delay from light onset was 4.0 ± 0.2 ms (Figure 5D). To rule out indirect connections, TTX (1 μM) was applied. TTX blocked the light-induced PSCs (Figures 5E–G). The combination of TTX and 4AP (1 mM) rescued the light-induced PSCs, which was again blocked by gabazine, but not by CNQX (Figures 5F,G). We found the mean response delay was 4.2 ± 0.2 ms (Figure 5H). We recorded 24 cells from 5 mice, and all cells (24/24, 100%) were inhibited by blue light stimulation (Figure 5I). Taken together, these results confirmed that DRN_5–HT_ neurons were directly innervated and inhibited by VTA_Gad67+_ neurons through GABA neurotransmission.

### DRN_Gad67+_ Neurons Are Also Inhibited by VTA_Gad67+_ Neurons

During the experimental recordings from YC-positive 5-HT neurons, we also recorded from YC-negative non–5-HT neurons. Interestingly, 3/22 non–5-HT neurons showed an inhibitory response to a 20-Hz blue light pulse (Supplementary Figure 2). Therefore, we assumed that some non–5-HT neurons also received inhibitory input from VTA_Gad67+_ neurons. In addition to 5-HT neurons, GABAergic neurons are also distributed throughout the DRN (Monti, 2010b). To elucidate the connection between VTA_Gad67+_ neurons and DRN_Gad67+_ neurons, we used Gad67-Cre mice and injected a Cre-dependent AAV expressing ChR2-eYFP into the VTA and another Cre-dependent AAV expressing tdTomato into the DRN to visualize GABAergic neurons in the DRN (Figure 6A). Immunohistochemical study confirmed that ChR2 was exclusively expressed in the VTA (Figure 6B). *In vitro* slice patch clamp recording from ChR2-expressing VTA_Gad67+_ neurons revealed that these neurons were depolarized by a 10-Hz blue light pulse, which also significantly increased the spontaneous firing frequency by 989 ± 319% compared with pre-stimulation values (Figures 6C–E). We stained DRN-containing slices with an anti-Gad67 antibody to label GABA neurons. Co-labeling of tdTomato and Gad67-positive cell bodies confirmed that GABA neurons expressed tdTomato (Figure 6F, white arrow), which was innervated by nerve terminals from VTA_Gad67+_ neurons (Figure 6F, white arrowheads). We then recorded from the tdTomato-expressing DRN_Gad67+_ neurons by stimulating VTA_Gad67+_ nerve terminals (Figure 6G). In whole-cell current clamp recordings from DRN_Gad67+_ neurons, we found that a 20-Hz blue light pulse also silenced them. However, the 20-Hz yellow light stimulation had no effect (Figures 6H,I). Next, to explore the connection patterns, we recorded from DRN_Gad67+_ neurons in whole-cell voltage clamp mode at a -60 mV holding potential. Activation of VTA_Gad67+_ nerve terminals induced PSCs in response to a 5-ms blue light pulse (Figures 6J,K). These light-induced PSCs were blocked by the combination of TTX (1 μM), 4AP (1 mM), and gabazine (10 μM), but not by TTX and 4AP (Figures 6J,K), confirming a monosynaptic GABAergic connection. The mean response delay was 3.7 ± 0.4 ms (Figure 6L). We recorded 36 cells from 7 mice, where 15 cells (41.7%) responded to blue light stimulation (Figure 6M). From these results, we concluded that a fraction of DRN_Gad67+_ neurons were also directly innervated and inhibited by VTA_Gad67+_ neurons.

### DRN_5–HT_ Neurons Are Inhibited by DRN_Gad67+_ Neurons

Through the experiments thus far, we found that both DRN_5–HT_ and DRN_Gad67+_ neurons were inhibited by VTA_Gad67+_ neurons. However, previous reports show that DRN_5–HT_ neurons are also inhibited by local GABAergic interneurons (Huang et al., 2017; Zhou et al., 2017). Thus, we aimed to reveal whether the inhibitory role of DRN_Gad67+_ neurons on DRN_5–HT_ neurons was the same in Gad67-Cre mice. To individually visualize DRN_Gad67+_ and DRN_5–HT_ neurons, we generated a bigenic mouse line, Gad67-Cre; Tph2-tTA. We then mixed and injected into the DRN two AAV viral vectors, Cre-dependent ChR2-eYFP and tTA-dependent TetO tdTomato (Figure 7A). We stained DRN-containing brain slices with an anti-Tph2 antibody to label 5-HT neurons. Co-labeling of tdTomato- and Tph2-positive cell bodies confirmed that 5-HT neurons expressed tdTomato (Figure 7B, white arrows), and they were also innervated by nerve terminals from local DRN_Gad67+_ neurons (Figure 7B, white arrowheads). To confirm ChR2 expression, we recorded from DRN_Gad67+_ neurons. A 10-Hz blue light pulse significantly increased the spontaneous firing frequency by 1161 ± 375% compared with pre-stimulation values (Figures 7C–E). We then recorded from the tdTomato-expressing DRN_5–HT_ neurons by stimulating the local DRN_Gad67+_ nerve terminals (Figure 7F). In whole-cell current clamp, we found that a 20-Hz blue light stimulation significantly reduced the firing frequency. However, a 20-Hz yellow light stimulation had no effect (Figures 7G,H). Next, to explore the connection patterns, we recorded from DRN_5–HT_ neurons in whole-cell voltage clamp mode at a -60 mV holding potential. Activation of DRN_Gad67+_ nerve terminals in the DRN induced PSCs in response to a 5-ms blue light pulse (Figure 7I). These light-induced PSCs were blocked by the combination of TTX (1 μM), 4AP (1 mM), and gabazine (10 μM), but not by TTX and 4AP (Figures 7I,J), confirming a monosynaptic GABAergic connection. The mean synaptic delay was 3.7 ± 0.4 ms (Figure 7K). We recorded 55 cells from 5 mice, where 14 cells (25.5%) responded to blue light stimulation (Figure 7L). Finally, we confirmed that a small fraction of DRN_5–HT_ neurons was also directly innervated and inhibited by local DRN_Gad67+_ neurons.

## Discussion

In this study, we discovered that the DRN receives dense projections from VTA_Gad67+_ neurons. In addition, we found that DRN_5–HT_ neurons were directly innervated and inhibited by not only VTA_Gad67+_ neurons but also by DRN_Gad67+_ neurons. Further, we showed that DRN_Gad67+_ neurons also receive monosynaptic inhibitory input from VTA_Gad67+_ neurons. *In vitro* recording from VTA_Gad67+_ neurons revealed that the cholinergic agonist CCh activated them, while DA, HA, and 5-HT inhibited them. However, application of the wake-promoting substances OX-A and NA had little or almost no effect on VTA_Gad67+_ neurons.

GAD65 and GAD67 are isoenzymes that are both critical for the synthesis of GABA, even though their expression patterns and mode of regulating synaptic plasticity are quite different (Esclapez and Houser, 1999; Lau and Murthy, 2012). GAD65, GAD67 and a vesicular GABA transporter (vGAT) are frequently used to target GABAergic neurons. Li et al. (2019) showed that rostral and caudal VTA GAD65-expressing neural populations had distinct projection patterns to their downstream targets. Also, there are some local inhibitory interneurons that exert indirect effects on neighboring dopaminergic or glutamatergic neurons. Chowdhury et al. (2019) reported that a subpopulation of vGAT-expressing neurons colocalized with GAD67-positive neurons in the VTA. Here, we used the Gad67-Cre mouse line to exclusively label the GAD67-expressing neuronal population in the VTA. The results we observed in our study might differ if a different Cre line is used to target GABAergic neurons.

VTA_Gad67+_ neurons receive major inhibitory and excitatory inputs from the bed nucleus of the stria terminalis, prefrontal cortex (PFC), lateral hypothalamic area (LHA), superior colliculus, nucleus accumbens (NAc), lateral habenula (LHb), DRN, and periaqueductal gray (PAG), as well as from local DA neurons (Bouarab et al., 2019). However, it is not clear how VTA_Gad67+_ neurons are regulated by neurotransmitters released from these input neurons. To clarify this, we locally applied substances that are directly or indirectly involved in the regulation of sleep/wakefulness to see the responses of the VTA_Gad67+_ neurons (Miller and O’Callaghan, 2006; Scammell et al., 2017; Holst and Landolt, 2018). Among the substances tested, 5-HT had a strong inhibitory effect on VTA_Gad67+_ neurons. Although it has been reported that non-DA neurons in the VTA showed both excitatory and inhibitory responses upon 5-HT application (Pessia et al., 1994), we found only inhibitory effect. Furthermore, we found that DRN_5–HT_ neurons were directly innervated and inhibited by VTA_Gad67+_ neurons. 5-HT mediated the inhibitory input from the DRN to the VTA_Gad67+_ neurons, a functional connection that could be involved in the promotion of wakefulness, since DRN_5–HT_ neurons are thought to be active during wakefulness. Although there has not been any report to date about the neural circuit between the VTA and tuberomammillary nucleus, HA showed a strong inhibitory effect on VTA_Gad67+_ neurons. Among four HA receptors, both H_3_ and H_4_ receptors are coupled with Gi G-protein (Panula et al., 2015). Thus, there is a possibility that our recording neurons might express H_3_ or H_4_ or both receptors if this is a direct effect. If this is indirect effect, H_1_ receptor mediated activation of inhibitory neurons is also possible. As we did not block the indirect effect from synaptic inputs of other neurons, we could not confirm whether the effect was direct or indirect. We need to do more experiments in future to explore pharmacological inspection. HA neurons in the tuberomammillary nucleus are also tonically active during wakefulness (Yu et al., 2014). This inhibition might also contribute to promoting wakefulness, similar to the effects imparted by DRN_5–HT_ neurons.

The release probability of NA is higher during wakefulness versus during sleep, but application of NA induced only a weak inhibition, or almost no significant effect, on VTA_Gad67+_ neurons. NA neurons in the LC are also known to be active during wakefulness. The differences in response patterns among monoaminergic neurotransmitters might be involved in the generation of different levels of wakefulness. Even though the basal forebrain and brainstem cholinergic neurons containing acetylcholine are involved in sleep/wakefulness, the VTA receives cholinergic innervation from only the brainstem pedunculopontine and laterodorsal tegmental nuclei (Oakman et al., 1995; Holmstrand and Sesack, 2011; Boucetta et al., 2014; Agostinelli et al., 2019). Kroeger et al. (2017) reported that activation of pedunculopontine cholinergic neurons increased NREM sleep, and we reported that activation of VTA_Gad67+_ neurons also increased NREM sleep (Chowdhury et al., 2019). Thus, since application of the cholinergic agonist CCh activated VTA_Gad67+_ neurons, this might suggest that the cholinergic inputs from pedunculopontine and laterodorsal tegmental nuclei regulate sleep/wakefulness through activation of VTA_Gad67+_ neurons.

It is not only the DA neurons in the VTA, but also those in the NAc and DRN that are critically involved in promoting wakefulness (Lu et al., 2006; Eban-Rothschild et al., 2016; Cho et al., 2017; Oishi et al., 2017; Luo et al., 2018). DA neurons in the VTA are inhibited by their local GABAergic neurons, but the functional relationship between DA neurons and GABAergic neurons in the VTA remains elusive. Here, we found that DA inhibited VTA_Gad67+_ neurons. Therefore, this inhibition of neighboring GABAergic neurons (VTA_Gad67+_) is a probable mechanism of DA neuron–induced wakefulness. We have reported that orexin neurons in the LHA receive direct inhibitory input from VTA_Gad67+_ neurons (Chowdhury et al., 2019). Moreover, orexin neurons mediate various physiological functions, including sleep/wakefulness regulation, energy homeostasis, reward seeking and drug addiction through their projections to the VTA (Xu et al., 2013). But surprisingly, we found that local application of OX-A failed to induce any effect in VTA_Gad67+_ neurons. Thus, the mutual interaction between orexin neurons in the LHA and VTA is unclear from our data, and more studies are needed to further understand the interaction.

VTA_Gad67+_ neurons have a wide range of projections throughout the whole brain, including to the PFC, CeA, NAc, LHb, ventral pallidus (VP), LHA, DRN, PAG, and local DA (Bouarab et al., 2019; Chowdhury et al., 2019). Virus-based retrograde and anterograde tracing studies have elucidated the anatomical locations of these projections. However, functional connections at the neural circuit level are still unclear. Downstream targets like NAc, LHb, and LHA receive inhibitory input from GABAergic neurons in the VTA (Brown et al., 2012; Root et al., 2014; Chowdhury et al., 2019). Our results are consistent with a recent report (Li et al., 2019) where the authors targeted rostral and caudal VTA GAD65-positive neurons and their functional connections with the GABAergic and 5-HT neurons of the DRN. Here, we confirmed again the functional connections between VTA_Gad67+_ neurons and DRN_Gad67+_ and DRN_5–HT_ neurons. Although we observed nerve terminals from VTA_Gad67+_ neurons in the CeA and LC by immunostaining and patch clamp experiments, we failed to observe any functional connections between VTA_Gad67+_ neurons and CeA or LC neurons. However, the number of recorded neurons was too small to unequivocally conclude that there are no such connections. Further neuroanatomical and electrophysiological experiments are required to verify any functional interactions between VTA_Gad67+_ neurons and those in the CeA and LC.

The DRN regulates numerous physiological functions by integrating inputs from the whole brain (Okaty et al., 2019). Optogenetic or chemogenetic activation of DRN_5–HT_ neurons can induce active wakefulness (Moriya et al., 2021) and increase depressive-like behavior (Teissier et al., 2015), but it halts spontaneous activity (Correia et al., 2017). On the contrary, 5-HT deficiency in the adult brain increases locomotor activity without inducing anxiety-like behavior (Whitney et al., 2016). Based on this, it is now clear that the DRN has subpopulations of 5-HT neurons (Huang et al., 2019). DRN_5–HT_ neurons receive both excitatory and inhibitory monosynaptic inputs from distal parts of the brain, like the mPFC (Geddes et al., 2016), LHb (Zhou et al., 2017), retinal ganglion cells (Huang et al., 2017), and caudal VTA (Li et al., 2019). Here, all the recorded Tph2-positive DRN_5–HT_ neurons received monosynaptic inhibitory input from VTA_Gad67+_ neurons. Apart from 5-HT neurons, another important neuronal subtype is GABAergic neurons in the DRN (Huang et al., 2019). These inhibitory neurons directly inhibit their neighboring DRN_5–HT_ neurons (Challis et al., 2013). Moreover, this kind of local inhibition is highly critical for 5-HT to be able to mediate numerous physiological functions (Huang et al., 2017; Li et al., 2019). Only a quarter of our recorded DRN_5–HT_ neurons received monosynaptic inhibition from their local DRN_Gad67+_ neurons. We also found that one half of our recorded DRN_Gad67+_ neurons were inhibited by VTA_Gad67+_ neurons. Thus, it is expected that VTA_Gad67+_ and DRN_Gad67+_ neurons control the activity of DRN_5–HT_ neurons through feedforward inhibition. Additional studies are required to clarify the precise regulatory mechanisms. Furthermore, it would be fascinating to explore the inhibitory role of the Gad67 population in DRN_5–HT_ neuron–regulated sleep/wakefulness and other physiological behaviors.

Taken together, we showed that VTA_Gad67+_ neurons were regulated by neurotransmitters involved in sleep/wakefulness regulation, such as 5-HT, DA, HA, and acetylcholine, but not by OX-A. VTA_Gad67+_ neurons integrated these inputs and inhibited DRN_5–HT_ neurons. This functional interaction between VTA_Gad67+_ and DRN_5–HT_ neurons might contribute to the understanding of the regulatory mechanisms of sleep/wakefulness.

## Data Availability Statement

The raw data supporting the conclusions of this article will be made available by the authors, without undue reservation.

## Ethics Statement

The animal study was reviewed and approved by Institutional Animal Care and Use Committees of the Research Institute of Environmental Medicine, Nagoya University, Japan (Approval numbers: R210096 and R210729).

## Author Contributions

SC and AY designed the experiments. SMR performed the experiments. DO, HY, SMR, YM, and AY contributed to the data analysis. SMR and AY wrote the manuscript. All authors contributed to the article and approved the submitted version.

## Conflict of Interest

The authors declare that the research was conducted in the absence of any commercial or financial relationships that could be construed as a potential conflict of interest.

## Publisher’s Note

All claims expressed in this article are solely those of the authors and do not necessarily represent those of their affiliated organizations, or those of the publisher, the editors and the reviewers. Any product that may be evaluated in this article, or claim that may be made by its manufacturer, is not guaranteed or endorsed by the publisher.

## References

[B1] Agostinelli L. J. Geerling J. C. Scammell T. E. (2019). Basal forebrain subcortical projections. *Brain Struct. Funct.* 224 1097–1117. 10.1007/s00429-018-01820-6 30612231 PMC6500474

[B2] Berndt A. Schoenenberger P. Mattis J. Tye K. M. Deisseroth K. Hegemann P. (2011). High-efficiency channelrhodopsins for fast neuronal stimulation at low light levels. *Proc. Natl. Acad. Sci. U.S.A.* 108 7595–7600. 10.1073/pnas.1017210108 21504945 PMC3088623

[B3] Bouarab C. Thompson B. Polter A. M. (2019). VTA GABA neurons at the interface of stress and reward. *Front. Neural Circuits* 13:78. 10.3389/fncir.2019.00078 31866835 PMC6906177

[B4] Boucetta S. Cisse Y. Mainville L. Morales M. Jones B. E. (2014). Discharge profiles across the sleep-waking cycle of identified cholinergic, GABAergic, and glutamatergic neurons in the pontomesencephalic tegmentum of the rat. *J. Neurosci.* 34 4708–4727. 10.1523/JNEUROSCI.2617-13.2014 24672016 PMC3965793

[B5] Brown M. T. Tan K. R. O’Connor E. C. Nikonenko I. Muller D. Luscher C. (2012). Ventral tegmental area GABA projections pause accumbal cholinergic interneurons to enhance associative learning. *Nature* 492 452–456. 10.1038/nature11657 23178810

[B6] Challis C. Boulden J. Veerakumar A. Espallergues J. Vassoler F. M. Pierce R. C. (2013). Raphe GABAergic neurons mediate the acquisition of avoidance after social defeat. *J. Neurosci.* 33 13978–13988, 13988a. 10.1523/JNEUROSCI.2383-13.2013 23986235 PMC3756748

[B7] Cho J. R. Treweek J. B. Robinson J. E. Xiao C. Bremner L. R. Greenbaum A. (2017). Dorsal raphe dopamine neurons modulate arousal and promote wakefulness by salient stimuli. *Neuron* 94 1205–1219.e8. 10.1016/j.neuron.2017.05.020 28602690

[B8] Chowdhury S. Yamanaka A. (2016). Optogenetic activation of serotonergic terminals facilitates GABAergic inhibitory input to orexin/hypocretin neurons. *Sci. Rep.* 6:36039. 10.1038/srep36039 27824065 PMC5099903

[B9] Chowdhury S. Matsubara T. Miyazaki T. Ono D. Fukatsu N. Abe M. (2019). GABA neurons in the ventral tegmental area regulate non-rapid eye movement sleep in mice. *Elife* 8:e44928. 10.7554/eLife.44928 31159923 PMC6548506

[B10] Cohen J. Y. Haesler S. Vong L. Lowell B. B. Uchida N. (2012). Neuron-type-specific signals for reward and punishment in the ventral tegmental area. *Nature* 482 85–88. 10.1038/nature10754 22258508 PMC3271183

[B11] Cools R. Roberts A. C. Robbins T. W. (2008). Serotoninergic regulation of emotional and behavioural control processes. *Trends Cogn. Sci.* 12 31–40. 10.1016/j.tics.2007.10.011 18069045

[B12] Correia P. A. Lottem E. Banerjee D. Machado A. S. Carey M. R. Mainen Z. F. (2017). Transient inhibition and long-term facilitation of locomotion by phasic optogenetic activation of serotonin neurons. *Elife* 6:e20975. 10.7554/eLife.20975 28193320 PMC5308893

[B13] Descarries L. Watkins K. C. Garcia S. Beaudet A. (1982). The serotonin neurons in nucleus raphe dorsalis of adult rat: a light and electron microscope radioautographic study. *J. Comp. Neurol.* 207 239–254. 10.1002/cne.902070305 7107985

[B14] Eagle D. M. Lehmann O. Theobald D. E. Pena Y. Zakaria R. Ghosh R. (2009). Serotonin depletion impairs waiting but not stop-signal reaction time in rats: implications for theories of the role of 5-HT in behavioral inhibition. *Neuropsychopharmacology* 34 1311–1321. 10.1038/npp.2008.202 19005464

[B15] Eban-Rothschild A. Rothschild G. Giardino W. J. Jones J. R. de Lecea L. (2016). VTA dopaminergic neurons regulate ethologically relevant sleep-wake behaviors. *Nat. Neurosci.* 19 1356–1366. 10.1038/nn.4377 27595385 PMC5519826

[B16] Erlander M. G. Tillakaratne N. J. Feldblum S. Patel N. Tobin A. J. (1991). Two genes encode distinct glutamate decarboxylases. *Neuron* 7 91–100. 10.1016/0896-6273(91)90077-d 2069816

[B17] Esclapez M. Houser C. R. (1999). Up-regulation of GAD65 and GAD67 in remaining hippocampal GABA neurons in a model of temporal lobe epilepsy. *J. Comp. Neurol.* 412 488–505. 10.1002/(sici)1096-9861(19990927)412:3<488::aid-cne8>3.0.co;2-6 10441235

[B18] Fields H. L. Hjelmstad G. O. Margolis E. B. Nicola S. M. (2007). Ventral tegmental area neurons in learned appetitive behavior and positive reinforcement. *Annu. Rev. Neurosci.* 30 289–316. 10.1146/annurev.neuro.30.051606.094341 17376009

[B19] Gately P. F. Poon S. L. Segal D. S. Geyer M. A. (1985). Depletion of brain serotonin by 5,7-dihydroxytryptamine alters the response to amphetamine and the habituation of locomotor activity in rats. *Psychopharmacology (Berl.)* 87 400–405. 10.1007/BF00432502 3936096

[B20] Geddes S. D. Assadzada S. Lemelin D. Sokolovski A. Bergeron R. Haj-Dahmane S. (2016). Target-specific modulation of the descending prefrontal cortex inputs to the dorsal raphe nucleus by cannabinoids. *Proc. Natl. Acad. Sci. U.S.A.* 113 5429–5434. 10.1073/pnas.1522754113 27114535 PMC4868450

[B21] Gossen M. Freundlieb S. Bender G. Muller G. Hillen W. Bujard H. (1995). Transcriptional activation by tetracyclines in mammalian cells. *Science* 268 1766–1769. 10.1126/science.7792603 7792603

[B22] Guan Y. Z. Ye J. H. (2010). Ethanol blocks long-term potentiation of GABAergic synapses in the ventral tegmental area involving mu-opioid receptors. *Neuropsychopharmacology* 35 1841–1849. 10.1038/npp.2010.51 20393452 PMC2904870

[B23] Hale M. W. Shekhar A. Lowry C. A. (2012). Stress-related serotonergic systems: implications for symptomatology of anxiety and affective disorders. *Cell. Mol. Neurobiol.* 32 695–708. 10.1007/s10571-012-9827-1 22484834 PMC3378822

[B24] Holmstrand E. C. Sesack S. R. (2011). Projections from the rat pedunculopontine and laterodorsal tegmental nuclei to the anterior thalamus and ventral tegmental area arise from largely separate populations of neurons. *Brain Struct. Funct.* 216 331–345. 10.1007/s00429-011-0320-2 21556793 PMC3255475

[B25] Holst S. C. Landolt H. P. (2018). Sleep-Wake Neurochemistry. *Sleep Med. Clin.* 13 137–146. 10.1016/j.jsmc.2018.03.002 29759265

[B26] Huang K. W. Ochandarena N. E. Philson A. C. Hyun M. Birnbaum J. E. Cicconet M. (2019). Molecular and anatomical organization of the dorsal raphe nucleus. *Elife* 8:e46464. 10.7554/eLife.46464 31411560 PMC6726424

[B27] Huang L. Yuan T. Tan M. Xi Y. Hu Y. Tao Q. (2017). A retinoraphe projection regulates serotonergic activity and looming-evoked defensive behaviour. *Nat. Commun.* 8:14908. 10.1038/ncomms14908 28361990 PMC5381010

[B28] Inutsuka A. Yamashita A. Chowdhury S. Nakai J. Ohkura M. Taguchi T. (2016). The integrative role of orexin/hypocretin neurons in nociceptive perception and analgesic regulation. *Sci. Rep.* 6:29480. 10.1038/srep29480 27385517 PMC4935841

[B29] Kanemaru K. Sekiya H. Xu M. Satoh K. Kitajima N. Yoshida K. (2014). In vivo visualization of subtle, transient, and local activity of astrocytes using an ultrasensitive Ca(2+) indicator. *Cell Rep.* 8 311–318. 10.1016/j.celrep.2014.05.056 24981861

[B30] Kroeger D. Ferrari L. L. Petit G. Mahoney C. E. Fuller P. M. Arrigoni E. (2017). Cholinergic, glutamatergic, and GABAergic neurons of the pedunculopontine tegmental nucleus have distinct effects on sleep/wake behavior in mice. *J. Neurosci.* 37 1352–1366. 10.1523/JNEUROSCI.1405-16.2016 28039375 PMC5296799

[B31] Lau C. G. Murthy V. N. (2012). Activity-dependent regulation of inhibition via GAD67. *J. Neurosci.* 32 8521–8531. 10.1523/JNEUROSCI.1245-12.2012 22723692 PMC3388776

[B32] Li Y. Li C. Y. Xi W. Jin S. Wu Z. H. Jiang P. (2019). Rostral and caudal ventral tegmental area GABAergic inputs to different dorsal raphe neurons participate in opioid dependence. *Neuron* 101 748–761.e5. 30638902 10.1016/j.neuron.2018.12.012

[B33] Li Y. Zhong W. Wang D. Feng Q. Liu Z. Zhou J. (2016). Serotonin neurons in the dorsal raphe nucleus encode reward signals. *Nat. Commun.* 7:10503. 10.1038/ncomms10503 26818705 PMC4738365

[B34] Lu J. Jhou T. C. Saper C. B. (2006). Identification of wake-active dopaminergic neurons in the ventral periaqueductal gray matter. *J. Neurosci.* 26 193–202. 10.1523/JNEUROSCI.2244-05.2006 16399687 PMC6674316

[B35] Luo Y. J. Li Y. D. Wang L. Yang S. R. Yuan X. S. Wang J. (2018). Nucleus accumbens controls wakefulness by a subpopulation of neurons expressing dopamine D1 receptors. *Nat. Commun.* 9:1576. 10.1038/s41467-018-03889-3 29679009 PMC5910424

[B36] Margolis E. B. Toy B. Himmels P. Morales M. Fields H. L. (2012). Identification of rat ventral tegmental area GABAergic neurons. *PLoS One* 7:e42365. 10.1371/journal.pone.0042365 22860119 PMC3409171

[B37] McDevitt R. A. Tiran-Cappello A. Shen H. Balderas I. Britt J. P. Marino R. A. M. (2014). Serotonergic versus nonserotonergic dorsal raphe projection neurons: differential participation in reward circuitry. *Cell Rep.* 8 1857–1869. 10.1016/j.celrep.2014.08.037 25242321 PMC4181379

[B38] Miller D. B. O’Callaghan J. P. (2006). The pharmacology of wakefulness. *Metabolism* 55 S13–S19. 16979420 10.1016/j.metabol.2006.07.007

[B39] Miyazaki K. Miyazaki K. W. Doya K. (2012). The role of serotonin in the regulation of patience and impulsivity. *Mol. Neurobiol.* 45 213–224. 10.1007/s12035-012-8232-6 22262065 PMC3311865

[B40] Monti J. M. (2010a). The role of dorsal raphe nucleus serotonergic and non-serotonergic neurons, and of their receptors, in regulating waking and rapid eye movement (REM) sleep. *Sleep Med. Rev.* 14 319–327. 10.1016/j.smrv.2009.10.003 20153670

[B41] Monti J. M. (2010b). The structure of the dorsal raphe nucleus and its relevance to the regulation of sleep and wakefulness. *Sleep Med. Rev.* 14 307–317. 10.1016/j.smrv.2009.11.004 20153669

[B42] Moriya R. Kanamaru M. Okuma N. Yoshikawa A. Tanaka K. F. Hokari S. (2021). Optogenetic activation of DRN 5-HT neurons induced active wakefulness, not quiet wakefulness. *Brain Res. Bull.* 177 129–142. 10.1016/j.brainresbull.2021.09.019 34563634

[B43] Mukai Y. Nagayama A. Itoi K. Yamanaka A. (2020). Identification of substances which regulate activity of corticotropin-releasing factor-producing neurons in the paraventricular nucleus of the hypothalamus. *Sci. Rep.* 10:13639. 10.1038/s41598-020-70481-5 32788592 PMC7424526

[B44] Nair-Roberts R. G. Chatelain-Badie S. D. Benson E. White-Cooper H. Bolam J. P. Ungless M. A. (2008). Stereological estimates of dopaminergic, GABAergic and glutamatergic neurons in the ventral tegmental area, substantia nigra and retrorubral field in the rat. *Neuroscience* 152 1024–1031. 10.1016/j.neuroscience.2008.01.046 18355970 PMC2575227

[B45] Nectow A. R. Schneeberger M. Zhang H. Field B. C. Renier N. Azevedo E. (2017). Identification of a brainstem circuit controlling feeding. *Cell* 170 429–442.e11. 10.1016/j.cell.2017.06.045 28753423

[B46] Niehaus J. L. Murali M. Kauer J. A. (2010). Drugs of abuse and stress impair LTP at inhibitory synapses in the ventral tegmental area. *Eur. J. Neurosci.* 32 108–117. 10.1111/j.1460-9568.2010.07256.x 20608969 PMC2908505

[B47] Nugent F. S. Kauer J. A. (2008). LTP of GABAergic synapses in the ventral tegmental area and beyond. *J. Physiol.* 586 1487–1493. 10.1113/jphysiol.2007.148098 18079157 PMC2375707

[B48] Nugent F. S. Penick E. C. Kauer J. A. (2007). Opioids block long-term potentiation of inhibitory synapses. *Nature* 446 1086–1090. 10.1038/nature05726 17460674

[B49] Oakman S. A. Faris P. L. Kerr P. E. Cozzari C. Hartman B. K. (1995). Distribution of pontomesencephalic cholinergic neurons projecting to substantia nigra differs significantly from those projecting to ventral tegmental area. *J. Neurosci.* 15 5859–5869. 10.1523/JNEUROSCI.15-09-05859.1995 7666171 PMC6577686

[B50] Ohmura Y. Tanaka K. F. Tsunematsu T. Yamanaka A. Yoshioka M. (2014). Optogenetic activation of serotonergic neurons enhances anxiety-like behaviour in mice. *Int. J. Neuropsychopharmacol.* 17 1777–1783. 10.1017/S1461145714000637 24834486

[B51] Oishi Y. Suzuki Y. Takahashi K. Yonezawa T. Kanda T. Takata Y. (2017). Activation of ventral tegmental area dopamine neurons produces wakefulness through dopamine D2-like receptors in mice. *Brain Struct. Funct.* 222 2907–2915. 10.1007/s00429-017-1365-7 28124114

[B52] Okaty B. W. Commons K. G. Dymecki S. M. (2019). Embracing diversity in the 5-HT neuronal system. *Nat. Rev. Neurosci.* 20 397–424. 10.1038/s41583-019-0151-3 30948838

[B53] Pan W. X. Brown J. Dudman J. T. (2013). Neural signals of extinction in the inhibitory microcircuit of the ventral midbrain. *Nat. Neurosci.* 16 71–78. 10.1038/nn.3283 23222913 PMC3563090

[B54] Panula P. Chazot P. L. Cowart M. Gutzmer R. Leurs R. Liu W. L. (2015). International union of basic and clinical pharmacology. XCVIII. Histamine receptors. *Pharmacol. Rev.* 67 601–655. 10.1124/pr.114.010249 26084539 PMC4485016

[B55] Pessia M. Jiang Z. G. North R. A. Johnson S. W. (1994). Actions of 5-hydroxytryptamine on ventral tegmental area neurons of the rat in vitro. *Brain Res.* 654 324–330. 10.1016/0006-8993(94)90495-2 7987681

[B56] Pignatelli M. Bonci A. (2015). Role of dopamine neurons in reward and aversion: a synaptic plasticity perspective. *Neuron* 86 1145–1157. 10.1016/j.neuron.2015.04.015 26050034

[B57] Polter A. M. Kauer J. A. (2014). Stress and VTA synapses: implications for addiction and depression. *Eur. J. Neurosci.* 39 1179–1188. 10.1111/ejn.12490 24712997 PMC4019343

[B58] Root D. H. Mejias-Aponte C. A. Zhang S. Wang H. L. Hoffman A. F. Lupica C. R. (2014). Single rodent mesohabenular axons release glutamate and GABA. *Nat. Neurosci.* 17 1543–1551. 10.1038/nn.3823 25242304 PMC4843828

[B59] Scammell T. E. Arrigoni E. Lipton J. O. (2017). Neural circuitry of wakefulness and sleep. *Neuron* 93 747–765. 10.1016/j.neuron.2017.01.014 28231463 PMC5325713

[B60] Schneeberger M. Parolari L. Das Banerjee T. Bhave V. Wang P. Patel B. (2019). Regulation of Energy Expenditure by Brainstem GABA Neurons. *Cell* 178 672–685.e12. 10.1016/j.cell.2019.05.048 31257028 PMC7481042

[B61] Schultz W. Dayan P. Montague P. R. (1997). A neural substrate of prediction and reward. *Science* 275 1593–1599. 10.1126/science.275.5306.1593 9054347

[B62] Seo C. Guru A. Jin M. Ito B. Sleezer B. J. Ho Y. Y. (2019). Intense threat switches dorsal raphe serotonin neurons to a paradoxical operational mode. *Science* 363 538–542. 10.1126/science.aau8722 30705194 PMC6777563

[B63] Shields A. K. Suarez M. Wakabayashi K. T. Bass C. E. (2021). Activation of VTA GABA neurons disrupts reward seeking by altering temporal processing. *Behav. Brain Res.* 410:113292. 10.1016/j.bbr.2021.113292 33836166

[B64] Simmons D. V. Petko A. K. Paladini C. A. (2017). Differential expression of long-term potentiation among identified inhibitory inputs to dopamine neurons. *J. Neurophysiol.* 118 1998–2008. 10.1152/jn.00270.2017 28701538 PMC5626910

[B65] Sun H. X. Wang D. R. Ye C. B. Hu Z. Z. Wang C. Y. Huang Z. L. (2017). Activation of the ventral tegmental area increased wakefulness in mice. *Sleep Biol. Rhythms* 15 107–115. 10.1007/s41105-017-0094-x 28386207 PMC5362655

[B66] Tan K. R. Yvon C. Turiault M. Mirzabekov J. J. Doehner J. Labouebe G. (2012). GABA neurons of the VTA drive conditioned place aversion. *Neuron* 73 1173–1183. 10.1016/j.neuron.2012.02.015 22445344 PMC6690362

[B67] Tanaka K. F. Matsui K. Sasaki T. Sano H. Sugio S. Fan K. (2012). Expanding the repertoire of optogenetically targeted cells with an enhanced gene expression system. *Cell Rep.* 2 397–406. 10.1016/j.celrep.2012.06.011 22854021

[B68] Teissier A. Chemiakine A. Inbar B. Bagchi S. Ray R. S. Palmiter R. D. (2015). Activity of raphe serotonergic neurons controls emotional behaviors. *Cell Rep.* 13 1965–1976. 10.1016/j.celrep.2015.10.061 26655908 PMC4756479

[B69] van Zessen R. Phillips J. L. Budygin E. A. Stuber G. D. (2012). Activation of VTA GABA neurons disrupts reward consumption. *Neuron* 73 1184–1194. 10.1016/j.neuron.2012.02.016 22445345 PMC3314244

[B70] Walther D. J. Peter J. U. Bashammakh S. Hortnagl H. Voits M. Fink H. (2003). Synthesis of serotonin by a second tryptophan hydroxylase isoform. *Science* 299:76. 10.1126/science.1078197 12511643

[B71] Weissbourd B. Ren J. DeLoach K. E. Guenthner C. J. Miyamichi K. Luo L. (2014). Presynaptic partners of dorsal raphe serotonergic and GABAergic neurons. *Neuron* 83 645–662. 10.1016/j.neuron.2014.06.024 25102560 PMC4779447

[B72] Whitney M. S. Shemery A. M. Yaw A. M. Donovan L. J. Glass J. D. Deneris E. S. (2016). Adult Brain serotonin deficiency causes hyperactivity, circadian disruption, and elimination of siestas. *J. Neurosci.* 36 9828–9842. 10.1523/JNEUROSCI.1469-16.2016 27656022 PMC5030349

[B73] Xiao J. Song M. Li F. Liu X. Anwar A. Zhao H. (2017). Effects of GABA microinjection into dorsal raphe nucleus on behavior and activity of lateral habenular neurons in mice. *Exp. Neurol.* 298 23–30. 10.1016/j.expneurol.2017.08.012 28843542

[B74] Xu T. R. Yang Y. Ward R. Gao L. Liu Y. (2013). Orexin receptors: multi-functional therapeutic targets for sleeping disorders, eating disorders, drug addiction, cancers and other physiological disorders. *Cell. Signal.* 25 2413–2423. 10.1016/j.cellsig.2013.07.025 23917208

[B75] Yu X. Li W. Ma Y. Tossell K. Harris J. J. Harding E. C. (2019). GABA and glutamate neurons in the VTA regulate sleep and wakefulness. *Nat. Neurosci.* 22 106–119. 10.1038/s41593-018-0288-9 30559475 PMC6390936

[B76] Yu X. Zecharia A. Zhang Z. Yang Q. Yustos R. Jager P. (2014). Circadian factor BMAL1 in histaminergic neurons regulates sleep architecture. *Curr. Biol.* 24 2838–2844. 10.1016/j.cub.2014.10.019 25454592 PMC4252164

[B77] Zell V. Steinkellner T. Hollon N. G. Warlow S. M. Souter E. Faget L. (2020). VTA Glutamate neuron activity drives positive reinforcement absent dopamine co-release. *Neuron* 107 864–873.e4. 10.1016/j.neuron.2020.06.011 32610039 PMC7780844

[B78] Zhou L. Liu M. Z. Li Q. Deng J. Mu D. Sun Y. G. (2017). Organization of functional long-range circuits controlling the activity of serotonergic neurons in the dorsal raphe nucleus. *Cell Rep.* 18 3018–3032. 10.1016/j.celrep.2017.02.077 28329692

[B79] Zhou Z. Liu X. Chen S. Zhang Z. Liu Y. Montardy Q. (2019). A VTA GABAergic neural circuit mediates visually evoked innate defensive responses. *Neuron* 103 473–488.e6. 10.1016/j.neuron.2019.05.027 31202540

