# Supplementary material for: Functional Interaction Between GABAergic Neurons in the Ventral Tegmental Area and Serotonergic Neurons in the Dorsal Raphe Nucleus
Source: Front Neurosci. 2022 May 19;16:877054. doi: 10.3389/fnins.2022.877054 (PMC9160575; doi:10.3389/fnins.2022.877054)
Supplement: Supplementary file 1 [file Data_Sheet_1.PDF]

Supplementary Material

1. Supplementary Figures

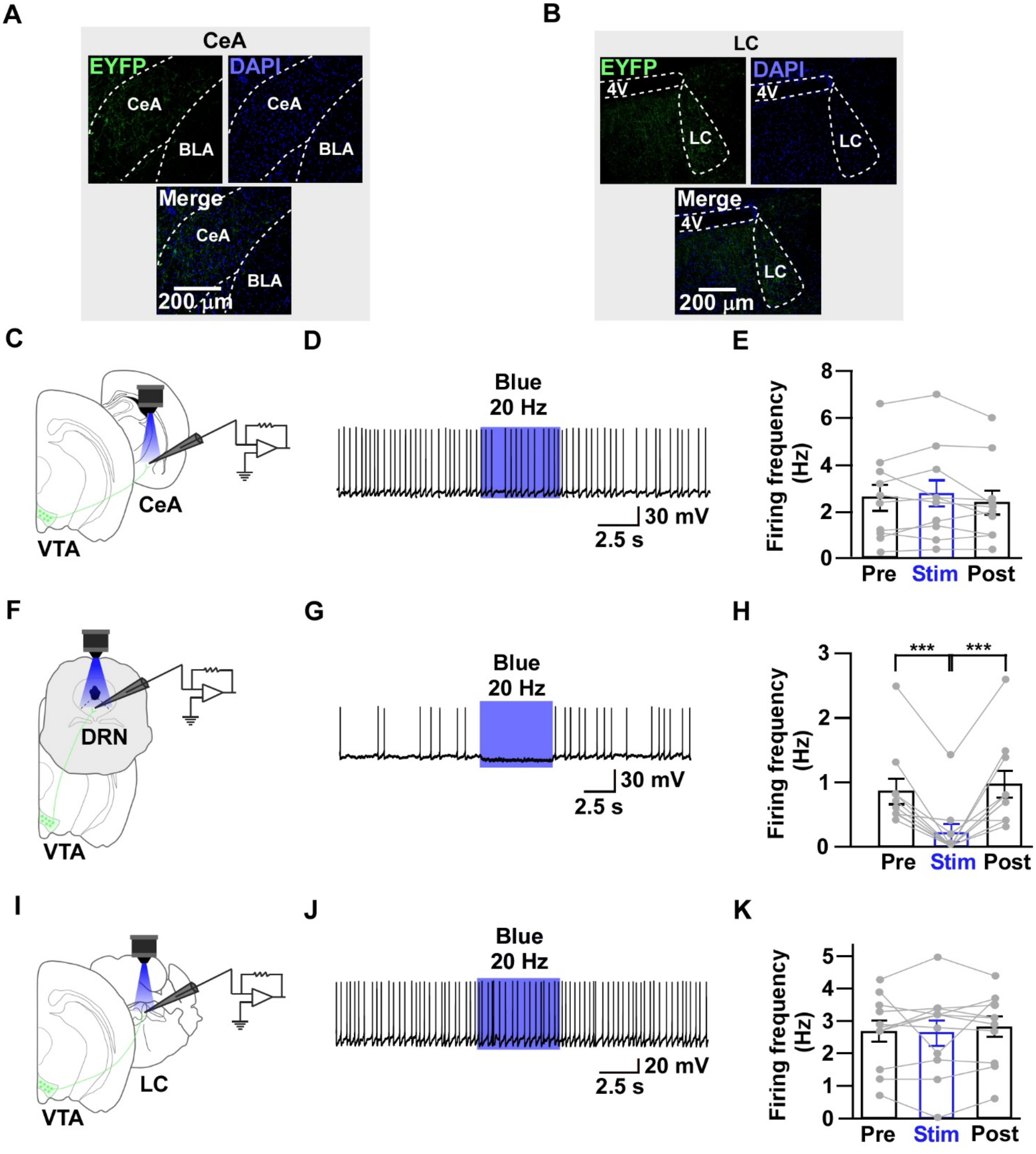

Supplemental Figure 1. Recording from neurons in the CeA, DRN and LC.

(A, B) Terminals of VTA_{Gad67+} neurons in the CeA and LC. Green (EYFP), blue (DAPI), white (merge). **(C, F, I)** Schematic of the whole-cell current clamp recording setup from the **(C)** CeA, **(F)** DRN and **(I)** LC neurons. **(D, G, J)** Representative traces of current clamp recordings from **(D)** CeA, **(G)** DRN, **(J)** LC neurons. VTA_{Gad67+} nerve terminals were activated by applying a 20-Hz stimulation of blue light (3.19 mW) for 5 s. **(E, H, K)** Summaries of **(D)**, **(G)** and **(J)**, respectively. Firing frequencies with blue light stimulation are compared with pre- and post-stimulation (CeA: n = 10 cells from 2 mice; DRN: n = 10 cells from 1 mouse; LC: n = 11 cells from 2 mice). Data are shown as means ± SEMs. All statistical analyses were made using one-way repeated measures ANOVA with Tukey's multiple comparison test. * $p<0.05$, ** $p<0.01$, *** $p<0.001$.

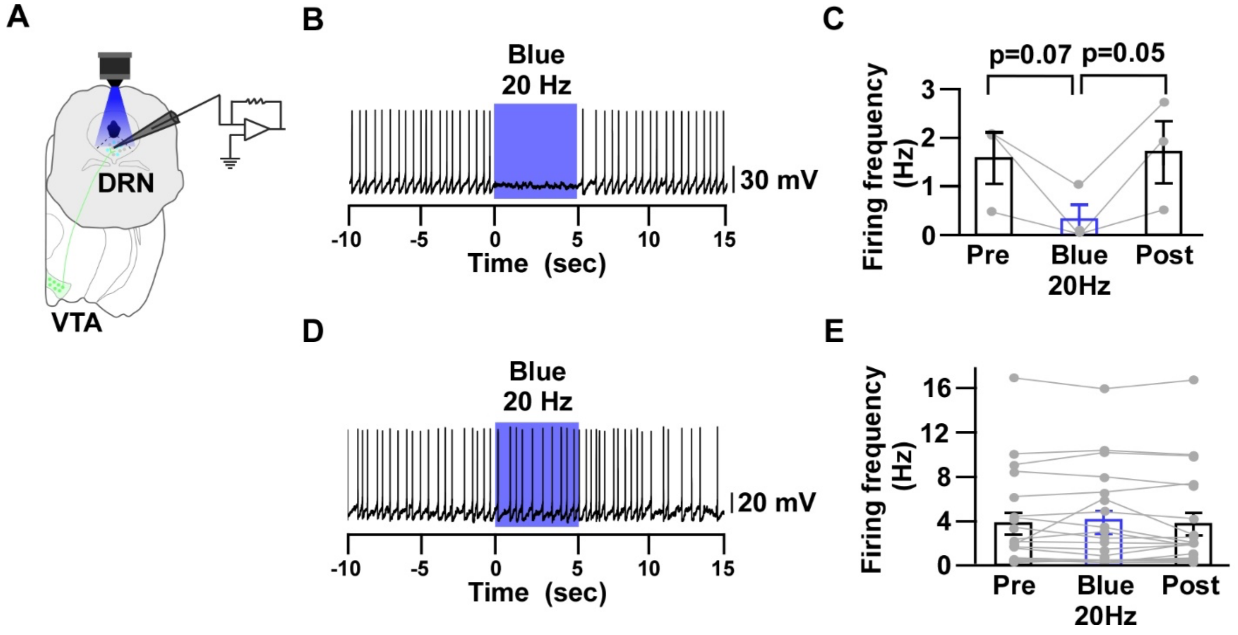

Supplemental Figure 2. Recording from YC-negative neurons in the DRN.

(A) Schematic of the setup for recording from YC-negative neurons in the DRN. **(B, D)** Representative traces of **(B)** inhibitory responses and **(D)** no responses from YC-negative neurons in the DRN. VTA_{Gad67+} nerve terminals were activated by applying a 20-Hz stimulation of blue light (3.19 mW) for 5 s. **(C, E)** Summaries of **(B)** and **(D)**, respectively. Firing frequencies with blue light stimulation are compared with pre- and post-stimulation. Data in panel **(E)** shows the combination of loose cell-attached and whole-cell current clamp recordings. **(C)** n = 3 cells from 4 mice; **(E)** n = 19 cells from 4 mice. Data are shown as means ± SEMs. All statistical analyses were made using a one-way repeated measures ANOVA with Tukey's multiple comparison test. * $p<0.05$, ** $p<0.01$, *** $p<0.001$.
